# Exposure to male‐dominated environments during development influences sperm sex ratios at sexual maturity

**DOI:** 10.1002/evl3.123

**Published:** 2019-06-27

**Authors:** Misha D. Lavoie, Jamie N. Tedeschi, Francisco Garcia‐Gonzalez, Renée C. Firman

**Affiliations:** ^1^ School of Biological Sciences (M092), Centre for Evolutionary Biology The University of Western Australia Crawley WA 6009 Australia; ^2^ Estacion Biológica de Doñana CSIC Sevilla Spain

**Keywords:** Developmental plasticity, house mice, male‐driven sex allocation, male–male competition, maternal effects, sex ratios

## Abstract

Different stages during development are important when it comes to phenotypic adjustments in response to external stimuli. Critical stages in mammals are the prenatal phase, where embryos are exposed to a milieu of sex steroid hormones, and the early‐postnatal phase, where littermates interact and experience their incipient social environment. Further, the postmaternal environment will influence the development of traits that are linked to reproductive success in adulthood. Accumulated evidence of male‐driven sex allocation establishes the currently untested hypothesis that the sperm sex ratio is a plastic trait that can be mediated to align with prevailing social conditions. Here, we used natural variation in the maternal environment and experimentally manipulated the postmaternal environment to identify the importance of these developmental phases on sperm sex ratio adjustments in wild house mice (*Mus musculus domesticus*). We found that male density in both environments was predictive of sperm sex ratios at sexual maturity: males from more male‐biased litters and males maturing under high male density produced elevated levels of Y‐chromosome‐bearing sperm. Our findings indicate that the sperm sex ratio is a variable phenotypic trait that responds to the external environment, and highlight the potential that these adjustments function as a mechanism of male‐driven sex allocation.

**Impact Summary**

Males have traditionally been dismissed as active players underlying sex ratio biases. However, recent evidence has indicated that male‐driven sex allocation is an emergent and important area of research. The most direct way by which fathers could allocate sex would be adjustments in the sperm sex ratio, but empirical proof for this mechanism as an arbiter of sex allocation remains elusive. Our study tests whether the sperm sex ratio is a plastic trait that can respond to prevailing social conditions. We used natural variation in the maternal environment and manipulated the postmaternal environment in mice, and provide evidence that exposure to high male density during development leads to higher levels of Y‐chromosome‐bearing sperm at sexual maturity.

It could be argued that offspring sex is the phenotypic trait that will have the greatest influence on an individual's inclusive fitness. Darwin (1871) first recognized that if an excess of one sex in a population was to occur, then “a tendency toward the equalization of the sexes would be brought about”, a theory formalized by Fisher (1930), who explained why equal investment in sons and daughters is an evolutionary stable strategy in terms of frequency‐dependent selection. Modifications of Fisher's assumptions later revealed how certain circumstances could lead to an adaptive bias in favor of one sex over the other (Hamilton 1967; Charnov 1982), for example via alterations of offspring sex ratios (i.e., defined as *n*_sons_/*n*_total offspring_). In some systems, offspring sex ratio adjustments that align with predictions have been routinely demonstrated (e.g., parasitoid wasps, spider mites) (Charnov et al. 1981; Macke et al. 2011). In other systems however, namely higher vertebrates, evidence of adaptive sex allocation is notoriously inconsistent and contradictory (Clutton‐Brock and Iason 1986). Nevertheless, facultative sex ratio adjustment in response to varied environmental or social conditions has been documented in different vertebrate species (Komdeur et al. 1997; Kruuk et al. 1999; Sheldon et al. 1999; Douhard et al. 2016; R.C. Firman unpubl. ms.). Consequently, genetic sex determination is no longer viewed as the all‐powerful constraint on sex allocation that it once was considered to be.

The social environment is expected to influence the way in which individuals allocate sex. For example, the production of male offspring under high male density conditions would be maladaptive as sons will be forced to compete with their nondispersing brothers, as well as unrelated local resident males, for access to females (Hamilton 1967). In the situation where females mate multiply, male–male competition will further extend to the postmating arena (Parker 2000; Parker et al. 2013). Under such conditions, however, competition among females is negligible. Rather, when male density is high, and daughters have a guarantee of high mate availability, the production of female offspring is expected to be advantageous. Studies of sex allocation in species that exhibit direct maternal control over offspring sex ratios (e.g., haplodiploid insects) have demonstrated how females will reduce sex ratios to minimize male–male competition and therefore maximize the fitness of their sons (Fellowes et al. 1999; Macke et al. 2011). There is strong evidence that local conditions influence individual sex allocation and population‐level sex ratio variation in vertebrates (e.g., Aars et al. 1995; Komdeur et al. 1997; Johnson et al. 2001; Silk and Brown 2008; Boulton and Fletcher 2015; Song et al. 2016), although these findings are more directly related to resource competition rather than mate competition per se (but see Saragusty et al. 2012; R.C. Firman unpubl. ms.).

Despite an overwhelming historical focus of the role of females in sex ratio manipulation, it is now recognized that both males and females can be effective arbiters of sex allocation (Edwards and Cameron 2014). In systems in which females are the heterogametic sex, such as birds, there is undisputed maternal control over the sex of ovulated eggs, with paternal contributions to offspring sex ratios being limited to maternal adjustments in relation to male phenotype (indirect) (Ellengren et al. 1996) or discriminatory posthatching care (direct) (Hasselquist and Kempenaers 2002). In mammals too, females—which typically incur a greater reproductive cost than males—have been at the core of adaptive sex allocation research. It is well established that female mammals can and will manipulate offspring sex ratios in relation to their own condition (Cameron 2004) or status (Clutton‐Brock et al. 1986), and/or in response to local conditions (Silk and Brown 2008; R.C. Firman unpubl. ms.). However, males will also benefit by exerting control over offspring sex ratios as means of maximizing their grand parentage (Edwards and Cameron 2014) and producing the sex that has the greatest opportunity for reproductive success (Gomendio et al. 2006; Saragusty et al. 2012; Douhard et al. 2016; Malo et al. 2017). As the mammalian heterogametic sex, the most feasible mechanism by which males can influence offspring sex ratios is through modifications of the sperm sex ratio (Edwards and Cameron 2014). Indeed, the assumption that equal proportions of X‐ and Y‐chromosome‐bearing sperm (CBS) are produced during spermatogenesis has been challenged by recent research demonstrating otherwise (e.g., Saragusty et al. 2012; Edwards et al. 2016a; Edwards and Cameron 2017; Malo et al. 2017). Male reproductive traits that are relevant to intrasexual selection have been shown to respond to the social environment in an adaptive manner (Firman et al. 2013, 2018; Ramm et al. 2015; André et al. 2018; Fisher et al. 2018). For example, male house mice that mature under a perceived risk of male–male competition invest more in growth (André et al. 2018; Firman et al. 2018) and sperm production (Firman et al. 2013, 2018) compared to males reared under noncompetitive conditions. Whether the competitive environment influences variation in sperm sex ratios is yet to be investigated.

The aim of this study was to determine whether sperm sex ratio is a plastic trait that can be mediated to align with prevailing social conditions, specifically in relation to variation in male density. Although individuals have the potential to respond to external stimuli throughout development, there are often critical life stages during ontogeny where the phenotype is particularly sensitive to the environmental variation. In mammals, the prenatal phase is a period during which the developing offspring are highly sensitive to external stimuli within the maternal environment. Two key factors during this life stage are (1) the connection to the mother and the influence of maternal hormones via placental transfer, and (2) exposure to the hormones produced by neighboring embryos (Kaiser and Sachser 2005). Indeed, the prenatal and early postnatal environments create the primary social conditions to which an individual is exposed. Following this, during sexual development, individuals are no longer exposed to the maternal environment but rather are exposed to a new suite of social conditions that can have important consequences for the development of reproductive traits. In this investigation, we optimized a qPCR molecular assay to provide an accurate method for quantifying sperm sex ratios. We then used natural variation in the maternal environment (i.e., conception to weaning) and experimentally manipulated the postmaternal environment (i.e., weaning to sexual maturity) to determine whether exposure to different densities of males during development influenced sperm sex ratios in adult house mice. In line with theory, we hypothesized that male‐dominated environments would favor the production of female offspring and therefore lead to an increase in the production of X‐CBS (i.e., lowered sperm sex ratios) (Hamilton 1967). We also measured a range of male traits known to be important for success in both pre‐ and postmating competition, namely body size (dominance and fighting ability), testes size (sperm production and a commonly used fertility index), and anogenital distance (AGD; a commonly used masculinity index). We expected to find that developing under high(er) male density would lead to males preparing for reproductive competition by investing in these traits.

## Methods

### EXPERIMENTAL MODEL AND SUBJECT DETAILS

Wild house mice (*Mus musculus domesticus*; *n* = 100) were captured and removed from Rat Island in the Abrolhos group off the coast of Western Australia (28°43′S 113°47′E) and outbred for three generations under standard laboratory conditions at the University of Western Australia. Common‐garden maintenance and breeding conditions were applied to eliminate potential factors that may induce phenotypic plasticity. Thus, mice were housed in standard cages (16 × 33 × 12 cm) and maintained at a constant temperature room (CTR; 24°C) on a reverse light–dark cycle (14:10), with food and water provided ad libitum. All animals experienced the same husbandry routine throughout the duration of the experiment.

### MATERNAL ENVIRONMENT

We used natural variation in the density of males within litters (i.e., litter sex ratio) to quantify differences in the maternal environment. To produce these experimental litters, unrelated male and female pairs (20) were mated under standard and controlled methods routinely performed in our lab (Firman and Simmons 2008, 2010). Matings were conducted during the dark phase under a red light (Firman and Simmons 2008). Females were checked regularly to detect estrus (Byers et al. 2012). When females were in estrus, matings were initiated with the introduction of a female into a male's cage. The female was then inspected half‐hourly for the presence of a mating plug. We used the presence of a mating plug as an indicator of a complete, successful mating event (Firman and Simmons 2008). After mating, each female was placed in a clean box with shredded paper for nesting and left undisturbed until parturition. Litter sex ratios were recorded at the time of birth and then left undisturbed until the time of weaning. The litters were weaned at three weeks of age, at which time mother's body mass and body mass of the experimental males (*n* = 2 males/family) were recorded.

### POSTMATERNAL ENVIRONMENT MANIPULATION

To manipulate the postmaternal social environment, we used similar methods of established protocols that we have used previously to induce phenotypic plasticity in the reproductive traits of house mice (Firman et al. 2013, 2018; André et al. 2018). We controlled the overall density of individuals that males were exposed to while manipulating their exposure to male and female pheromones (Firman and Simmons 2013). As house mice are able to recognize conspecifics via individually distinct scent signals (Hurst and Beynon 2004), our methods ensured that males experienced one of two different social environments during their sexual development (Firman and Simmons 2013). Brothers were used across treatments to control for family‐derived variation. The experimental males were reared in either a high male density (*n* = 20) or high female density (*n* = 20) environment. The different social environments were created by housing males in standard cages on metal racks in two separate CTRs. In one CTR, the high male density environment, focal males were reared from three to 15 weeks within close proximity to 25 nonfocal males, consisting of 10 sexually mature males and 15 males of the same age (Fig. S1A). Focal males were rotated through the rack positions on a regular schedule. Twice a week, each focal male was exposed to 15 g of soiled chaff from 10 nonfocal sexually mature males. Once a fortnight, each focal male experienced a “male encounter.” Thus, each focal male was released into a large, plastic opaque tub (49 × 74 × 41 cm) containing two sexually mature, nonfocal males (Fig. S1A). The nonfocal males remained housed in their cages for the duration of the encounter. Thus, the focal males roamed freely in the tub for 30 min but could only interact with the nonfocal males through the wire cage lids. The focal males were exposed to different nonfocal males in each encounter. To ensure normal reproductive development, the focal males were periodically exposed to soiled chaff from a sexually mature female.

A high female density environment was established in a second CTR. Here, males were reared from the age of three to 15 weeks within close proximity to 25 female mice (Fig. S1B), consisting of 10 sexually mature female mice and 15 females of the same age. As in the high male density environment, the males in the high female density environment were rotated through the rack positions on a regular schedule. Twice a week, each male was exposed to 15 g of soiled chaff from 10 sexually mature females. The males experienced fortnightly “female encounters,” which were conducted as described above but with sexually mature females, that is, the males were able to interact through wire cage lids with sexually mature females for 30 min (Fig. S1B). The treatments were swapped between the two CTRs midway through the experiment. The experimental males were exposed to the high male or high female density conditions until sexual maturity at which time they were euthanized via cervical dislocation (males aged between 104 and 107 days).

### ANATOMICAL MEASUREMENTS AND EPIDIDYMAL SPERM ISOLATION

Immediately following euthanasia, body mass, body length, AGD, and testes mass were recorded. The epididymis was dissected to extract sperm according to published protocols routinely performed in our laboratory (Firman and Simmons 2010; Firman et al. 2013). Specifically, both the left and right caudal epididymides were incised, placed in 1 mL of human tubal fluid (HTF), and incubated at 37°C under 5% CO_2_. Following an initial 10‐min incubation period, which allowed the sperm to swim into the medium, the epididymal tissue was removed from the HTF and the sperm suspension was incubated for a further 50 min (37°C, 5% CO_2_). Following incubation, aliquots of the sperm suspension (× 2 10 µL aliquots per sample) were removed and, with a CEROS computer‐assisted sperm analyzer (CASA; Hamilton Thorne, version 10), were used to measure epididymal sperm concentration. The sperm suspension was centrifuged for 10 min at 14,000 rpm, washed in 500 µL of Tris‐EDTA buffer solution, and recentrifuged for 5 min at 14,000 rpm to pellet the sperm and then resuspended in 200 µL of Tris‐EDTA buffer solution and stored at –20°C.

### SPERM DNA EXTRACTION AND qPCR QUANTIFICATION OF SPERM SEX RATIOS

Genomic DNA was extracted from pooled sperm samples by Chelex‐100 (Silva et al. 2014). Briefly, 25 µL of thawed sperm solution was added to 200 µL Chelex‐100 resin (5%) buffer and digested with 20 µL Proteinase K (20 mg/mL) and 7.6 µL DTT (31 mM) for 45 min at 56°C. Following digestion, proteinase K enzyme was deactivated with 8‐min incubation at 95°C. Samples were centrifuged for 3 min at 10,500 rpm to separate the DNA‐rich supernatant from cellular proteins. The supernatant was quantified by a NanoDrop® Spectrophotometer (ND1000, Thermo Fisher Scientific, Australia). All samples were standardized to 100 ng/µL by dilution with nuclease‐free water and stored at –20°C before quantitative real‐time polymerase chain reaction (qPCR).

Sperm sex ratio is routinely quantified by qPCR in domestic livestock (Tretipskul et al. 2011; Maleki et al. 2013; Khamlor et al. 2014). Here, we modified and optimized an absolute quantification qPCR protocol to measure the proportion of Y‐CBS in the sperm samples of house mice. To validate our method, we first tested the specificity of our mouse‐specific Taqman probes (X‐CBS: *G6pd2*; Y‐CBS: *Sry*) on sperm samples of house mice that had been reared under common‐garden conditions (*n* = 6). A five‐point twofold dilution series (from 100 to 6.25 ng/µL) was run through singleplex qPCRs to generate standard curves for each gene to determine primer binding efficiency (Pfaffl 2001; Rasmussen 2001). The primer efficiencies and goodness of fit for these curves (*G6pd2*: E = 2.00 or 99.7%, *r* ^2^ = 0.998; *Sry*: E = 2.02 or 101.7%, *r* ^2^ = 0.978) indicated that the optimal DNA concentration for detecting the proportion of Y‐ and X‐CBS was 100 ng/µL (common‐garden males: mean ± SE proportion Y‐CBS = 0.509 ± 0.001). For the experimental sperm samples, we amplified a standard concentration of 100 ng/µL of DNA for the *G6pd2* and *Sry* genes in triplicate 10 µL singleplex reactions with 1 µL DNA template, 5.0 µL Taqman Fast Advanced Master Mix [2×], 0.5 µL Taqman probe [20×], and 3.5 µL nuclease‐free water on a StepOnePlus^TM^ thermocycler (Applied Biosystems, Australia) with the following cycling conditions: incubation at 50°C for 2‐min and polymerase activation at 95°C for 20 sec, followed by 40 cycles of 95°C for 1 sec and 60°C for 20 sec. The fluorescent signals captured at the end of each amplification cycle produced the threshold cycle (*Ct*; i.e., the number of PCR cycles required for the fluorescence signal to cross a threshold line, which is inversely proportional to the amount of nucleic acid present in the sample). The repeatability of the triplicate qPCR assays was very high (*G6pd2* gene: *R* = 0.99, SE = 0.005, *P* ≪ 0.0001; *Sry* gene: *R* = 0.99, SE = 0.0008, *P* ≪ 0.0001) (Becker 1984). For quality control, we applied a 0.3 *Ct* standard deviation threshold for triplicate samples (i.e., *Ct* values that were out of this range were excluded from a replicate group). The mean of the replicate *G6pd2* gene and *Sry* gene *Ct* values for each sperm sample was used for calculating the proportion of X‐ and Y‐CBS (Parati et al. 2006). The proportion of Y‐CBS was calculated from the ratio between the quantities of X‐ and Y‐CBS using the following equation (Parati et al. 2006; Puglisi et al. 2006):

proportion Y-CBS=n/(n+1),

where *n* = *Ct*_Y‐CBS_/*Ct*_X‐CBS_.

For each sample, the number of X‐ and Y‐CBS was calculated using (1) the proportion measured in the qPCR assay, (2) the known volume of sperm suspension required for the qPCR assay (i.e., to a achieve standard concentration of 100 ng/µL of DNA), and (3) overall sperm concentration that was measured at the time of sperm isolation.

### STATISTICAL ANALYSIS

All statistical analyses were conducted in R version 3.5.1 (R Core Team 2018). We ran linear mixed models (LMM) in our analyses of the anatomical traits (body mass at weaning, body length and mass at sexual maturity, AGD, and testes mass) and sperm concentration, and generalized linear mixed models (GLMM) in the analyses of sperm sex ratios. In all cases, family identity was included as a random effect in the models. All models were initially fitted using the function *lmer* (LMMs) or *glmer* (GLMMs) implemented within the package *lme4* (Bates et al. 2015). Subsequently, for GLMMs we used penalized quasi‐likelihood by running the function *glmmPQL* from the *MASS* package to account for issues of data dispersion (Venables and Ripley 2002). We ran GLMMs with binomial distribution of errors using the command *cbind* so that the response variable contained information about the numbers of X‐ and Y‐CBS leading to the sperm sex ratio value in each sample.

We were interested in the effect of both the maternal and postmaternal social environment on the development of male traits. Thus, litter sex ratio (i.e., *n*_sons_/*n*_total litter size_) and treatment (i.e., high male density or high female density environment) were included in the models. Because an association between litter size and litter sex ratio could generate spurious correlations between litter sex ratio and our responses variables, prior to running our analyses we confirmed that there was no relationship between litter size and litter sex ratio within our data set (Fig. S2). Because maternal condition influences growth rate during development, mother's body mass was included as a covariate in the LMMs testing (1) body mass at weaning, (2) body length at sexual maturity, and (3) body mass at sexual maturity. To control for differences due to body size, body length was included as a covariate in the LMM testing testes mass and body mass was included as a covariate in the LMM testing AGD (our chosen covariates were based on the fact that body mass was not independent of testes mass and body length was not independent of AGD). We were interested in testing for differences in epididymal sperm concentration independent of testes size, and thus included testes mass as a covariate in this LMM. All the covariates were mean centered to improve the interpretability of regression coefficients, as recommended by Schielzeth (2010). Two outliers in testes mass due to abnormal testes development were excluded from the analyses; one male had abnormally large testes (+2SD from the mean) and one male had abnormally small testes (−2SD from the mean). Likewise, there were single outliers among the sperm concentration and sperm sex ratio data. These data were abnormally high (+2SD from the mean), and thus removed from the corresponding statistical models.

All two‐ and three‐way interaction terms were nonsignificant and consequently removed from the models (see Tables S1 and S2). Significance of the fixed effects in the LMMs was calculated using maximum likelihood and Wald tests, using the function ANOVA (*car* package), whereas parameter estimates were calculated using restricted maximum likelihood (REML) as recommended (Zuur et al. 2009). Significance of the fixed effects in the GLMMs was calculated with *t*‐tests using the *MASS* package. Visual inspection of diagnostic plots (qqplots and plots of the distribution of the residuals against fitted values) was checked to validate the models.

## Results

### ANATOMICAL TRAITS AND EPIDIDYMAL SPERM CONCENTRATION

**Figure 1:**
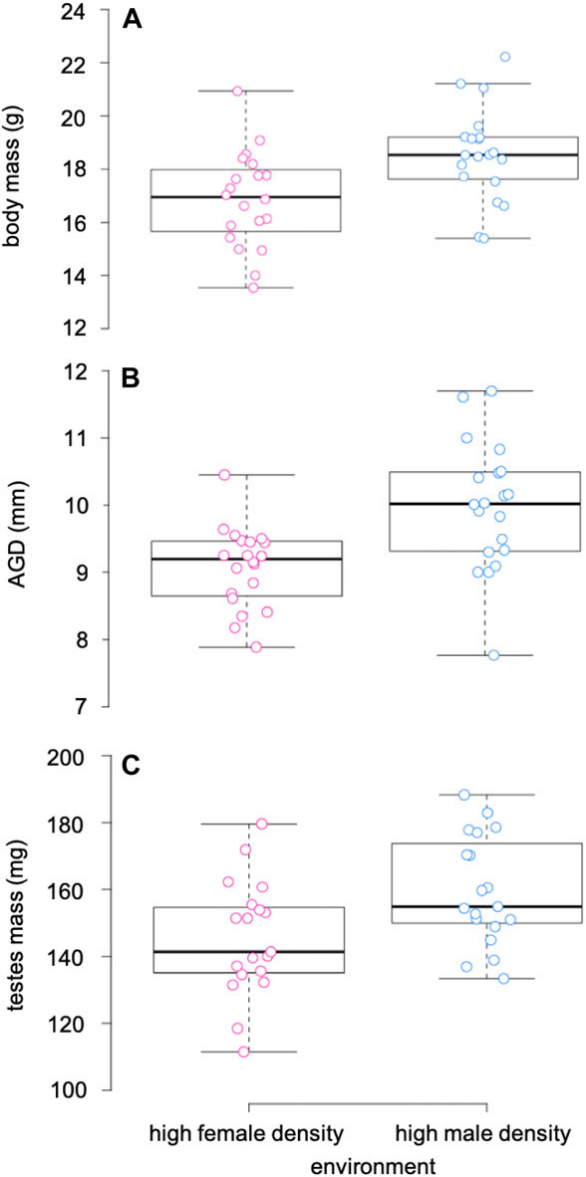
The postmaternal social environment influences body size, anogenital distance (AGD), and testes size in male house mice. Body mass (A), AGD (B), and testes mass (C) were greater among individuals reared in a high male density environment (blue points) compared to individuals reared in a high female density environment (pink points).

The only predictor of body mass at weaning age was mother's body mass, whereby larger females weaned larger sons (Table 1). Males did not differ in body mass prior to treatment (mean ± SE: male environment = 9.7 ± 1.6 g, female environment = 9.5 ± 1.4 g), however males exposed to the male environment during development reached a significantly larger size (mass: 18.5 ± 1.7 g; length: 74.0 ± 0.9 mm) than males exposed to the female environment (mass: 16.9 ± 1.8 g; length: 72.2 ± 0.8 mm) at sexual maturity (Table 1; Fig. 1A). Litter sex ratio also accounted for variation in body length; body length increased with an increasing proportion of males in the litter (Table 1). There was a significant treatment effect on both AGD and testes mass after controlling for body size; males from the male environment had both longer AGDs (9.98 ± 0.2 mm) and larger testes (159.6 ± 3.7 mg) than males from the female environment (AGD = 9.08 ± 0.1 mm; testes = 145.3 ± 3.9 mg) (Table 1; Fig. 1B, C). After controlling for testes mass, males reared in the male (12.62 ± 1.4 × 10^6^) and female (10.87 ± 1.3 × 10^6^) environment did not differ in epididymal sperm concentration (Table 1; Fig. S3).

**Table 1:** Linear mixed models (LMMs) on the effect of the social environment and litter sex ratio on anatomical traits of male house mice

Fixed effects	Estimate	±SE	Type II, Wald χ^2^	df	*P*‐value
Body mass: weaning					
Intercept	9.519	0.297			
Treatment	0.208	0.198	1.152	1	0.283
Litter sex ratio	–1.517	1.320	1.554	1	0.213
Mother's body mass	0.215	0.092	6.394	1	**0.011**
Body mass: maturity					
Intercept	16.855	0.367			
Treatment	1.692	0.510	11.810	1	**<0.001**
Litter sex ratio	1.341	1.248	1.332	1	0.248
Mother's body mass	0.231	0.087	8.103	1	**0.004**
Body length: maturity					
Intercept	72.162	0.767			
Treatment	1.858	0.960	3.938	1	**0.047**
Litter sex ratio	5.671	2.823	4.750	1	**0.029**
Mother's body mass	0.344	0.197	3.605	1	0.058
AGD					
Intercept	9.209	0.178			
Treatment	0.639	0.265	6.446	1	**0.011**
Litter sex ratio	–0.716	0.568	1.764	1	0.184
Body mass	0.156	0.070	5.606	1	**0.018**
Testes mass					
Intercept	147.289	3.732			
Treatment	9.900	4.557	5.276	1	**0.022**
Litter sex ratio	15.956	11.012	2.346	1	0.126
Body length	2.270	0.633	14.403	1	**<0.001**
Epididymal sperm concentration					
Intercept	11.897	1.502			
Treatment	0.318	1.556	0.050	1	0.824
Litter sex ratio	–2.570	6.190	0.202	1	0.653
Testes mass	0.096	0.060	2.919	1	0.088

*P*‐values in bold are significant at <0.05. Family ID was included as a random effect in all LMMs. High female density treatment level is the reference level for the treatment factor. Full models that include the two‐ and three‐way interaction terms are presented in Table S1. AGD, anogenital distance.

### SPERM SEX RATIO

**Figure 2:**
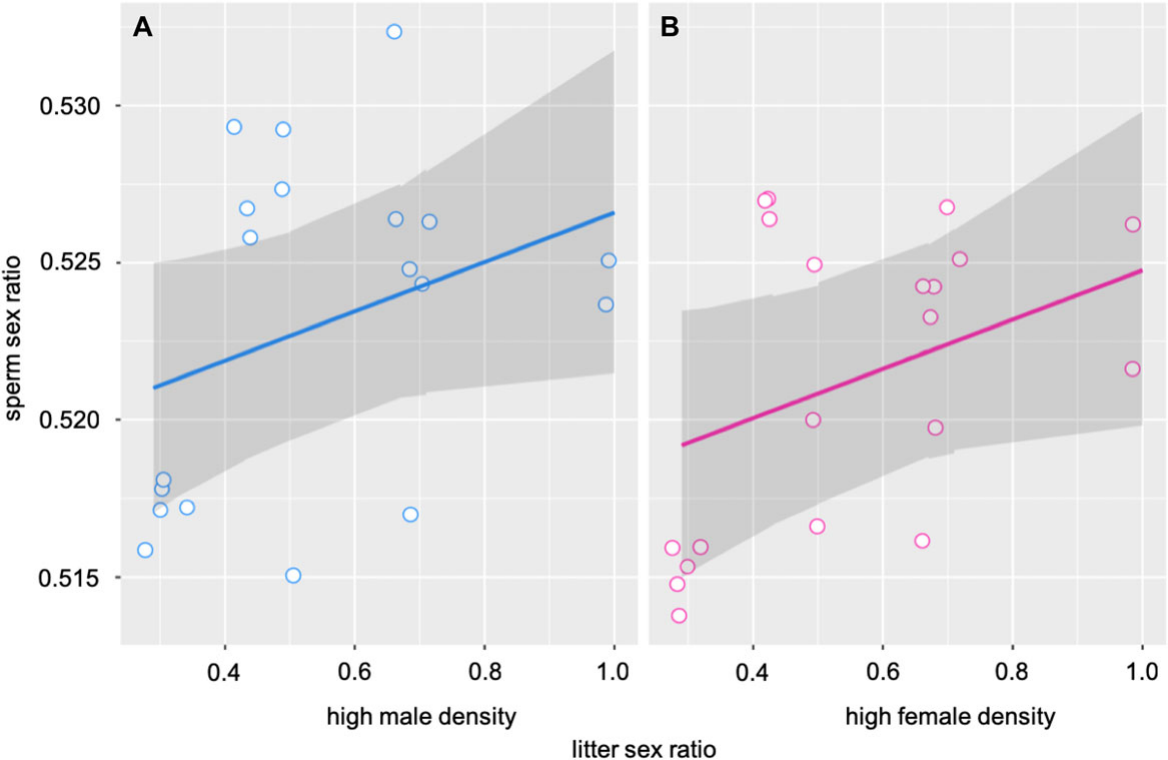
The developmental social environment influences sperm sex ratios in house mice. Sperm sex ratios were higher (i.e., greater proportions of Y‐CBS sperm) among individuals reared in a high male density environment (A) compared to individuals reared in a high female density environment (B), and sperm sex ratios increased with an increasing proportion of males in the litter (A and B). The figure shows the observations (blue and pink circles), the prediction from the model in the scale of the response variable (blue and pink solid lines), and the 95% CI from the bootstrapped sample (shadowed area) (the model prediction lines calculated with bootstrapping [*n* = 10,000] are not visible as they lie underneath the solid lines). We calculated the effect sizes for the difference in raw means (and associated standard deviations), using the package *compute.es*, or for the difference in the means from the model (estimated marginal means), and associated standard errors, using the package *emmeans* and online calculators. The effect size for the difference in sperm sex ratio between groups was medium (i.e., d [95% CI] = 0.36 [–0.29, 1.02] and d [95% CI] = 0.39 [–0.24, 1.03] calculated using raw means or means from GLMM model, respectively), although with corresponding wide CIs, which is most likely a consequence of small sample size.

Our analysis revealed that both treatment and litter sex ratio accounted for variation in sperm sex ratios (Table 2; Fig. 2A, B). Males reared in a male environment produced higher proportions of Y‐CBS compared to males reared in a female environment, and males from more male‐biased litters produced higher proportions of Y‐CBS (Fig. 2). Further, we explored the relationship between testes mass and the proportion of Y‐CBS as a possible mechanism accounting for variation in sperm sex ratio at sexual maturity by including testes mass as a covariate in our model. The GLMM revealed that testes mass did not explain any variation in sperm sex ratio (Table 2).

**Table 2:** Generalized linear mixed model (GLMM) using a binomial distribution of errors to assess the effect of the social environment and litter sex ratio on sperm sex ratios of house mice

Fixed effects	Estimate	±SE	*t*	df	*P*‐value
Without testes mass					
Intercept	0.086	0.004			
Treatment	0.007	0.003	2.644	18	**0.017**
Litter sex ratio	0.039	0.018	2.205	18	**0.041**
With testes mass					
Intercept	0.086	0.004			
Treatment	0.007	0.003	2.241	15	**0.041**
Litter sex ratio	0.035	0.018	1.978	18	0.064
Testes mass	0.000	0.000	0.813	15	0.429

Family ID was included as a random effect in the models. High female density treatment level is the reference level for the treatment factor. *p*‐values in bold are significant at <0.05.

## Discussion

Our results confirm that social cues perceived during development in both the maternal and postmaternal environment can have important and varying effects on male reproductive traits. We discovered that both the maternal and postmaternal environments influence sperm sex ratios at sexual maturity. Our study thus suggests that the sperm sex ratio is a malleable phenotypic trait and adds to accumulating evidence that the mechanistic basis of male‐driven sex allocation in mammals is an adjustment in the ratio of X‐ to Y‐CBS.

In this investigation on house mice, we uncovered the novel result that exposure to both pre‐ and postnatal male dominated environments resulted in the production of greater proportions of Y‐CBS. These findings contradict the expected bias toward the production of daughters (X‐CBS) under local mate competition theory (Hamilton 1967), but do provide compelling support for the male fertility hypothesis of paternal sex allocation (Gomendio et al. 2006). Existing evidence in support of this hypothesis comes from positive correlations between fertility parameters and the proportion of sons produced. For example, among studies of humans that have used the time taken to conceive as a proxy for (in)fertility, there is a general trend that subfertile couples disproportionately produce female offspring (Weijin and Olsen 1996; James 2008; but see Smits et al. 2005). However, one limitation in human studies is the inability to disentangle male‐ and female‐derived effects. An investigation of red deer retained natural variation in male fertility, but reduced variation in offspring sex allocation in relation to male quality, maternal condition, and the timing of conception by artificially inseminating females that were in optimal physical condition (Gomendio et al. 2006). It was reported that male red deer that induced higher pregnancy rates were also more likely to sire sons. The authors postulated that a possible mechanism explaining this phenomenon was that the ejaculates of fertile males contained higher proportions of Y‐CBS (Gomendio et al. 2006), a relationship that has since been documented in a subpopulation of infertile men (Eisenberg et al. 2012). Here, we report no difference in the number of sperm stored in the epididymis among males reared in different social environments, although we do report differences in testes size, which is a commonly used index for sperm production rates and male fertility (Møller 1989). Our analysis produced a nonsignificant result for the relationship between testes size and the proportion of Y‐CBS. However, considering that the current investigation was not specifically designed to test this hypothesis, we are hesitant to rule out the possibility that an adjustment in sperm sex ratio is the underlying mechanism accounting for the relationship between male fertility and the production of male offspring.

Male fertility is often advertized by the size or elaboration of sexual characters that females use to assess their quality (Sheldon 1994; Malo et al. 2005) (although see Pizzari et al. 2004). When sons inherit attractiveness traits from their fathers, and these traits have greater influence on the fitness of sons than on daughters, a sex ratio bias toward male offspring is adaptive (Ellengren et al. 1996). The benefits to females of male‐biased offspring production in relation to paternal attractiveness have been demonstrated in different vertebrate taxa (Ellengren et al. 1996; Pilastro et al. 2002). However, these benefits can also extend to the postmating arena in terms of the inheritance of traits that are important for success in sperm competition. For example, if more fertile males produce highly competitive Y‐CBS, then sperm competition dynamics would exacerbate the already existing male‐biased sex ratios. In house mice, increased body size is linked to enhanced fighting ability and dominance status, and therefore contributes to success in premating competition, whereas increased sperm production is critical to competitive fertilization success (Firman and Simmons 2011; Cunningham et al. 2013). Moreover, females may exercise mechanisms of cryptic female choice (CFC) to further skew sex ratios in favor of male offspring (Firman et al. 2017), for example via different immune responses to X‐ and Y‐CBS in the oviduct (Alminana et al. 2014). In our future research, we will explore the role of CFC in adaptive sex allocation and specifically test the male fertility hypothesis of paternal sex allocation (Gomendio et al. 2006).

We found that the density of male siblings in the maternal environment influenced sperm sex ratios at sexual maturity. It is possible that sons born to mothers that are genetically predisposed to producing male‐biased litters inherit a predisposition toward producing Y‐CBS. In this case, we may expect sperm sex ratios to be prenatally fixed. However, this explanation juxtaposes the observed response to the postmaternal environment. In placental mammals, the prenatal social environment can vary considerably, both in terms of the overall and relative density of male and female offspring, as well as in relation to the sex of neighboring embryos. Indeed, because sexual differentiation in mammals is largely influenced by androgens early in development, intrauterine position can have lasting effects on an individual's reproductive development (Ryan and Vandenbergh 2002). For example, females that develop between two males (2M) tend to show masculinized anatomical (including longer AGDs), physiological, and behavioral traits compared to females that develop between two females (0M) (reviewed Ryan and Vandenbergh 2002). Further, in different rodent species it has been shown that 2M males have larger testes and scent mark more frequently than 0M males (van der Hoeven et al. 1992; Clark et al. 1993). These effects of the intrauterine environment have been postulated to be due to the diffusion of testosterone from two adjacent brothers.

The effect of litter sex ratio on the proportion of Y‐CBS could be explained by variation in exposure to prenatal testosterone levels, for example due to differences in male density per se or due to higher proportions of 2M males with increasing litter sex ratios. Indeed, prenatal exposure to higher levels of testosterone may be the underlying mechanism accounting for the observed response of an increase in male body length with increasing litter sex ratio. Interestingly, however, the lack of an effect of the maternal environment on body mass, testes size, and AGD at sexual maturity is consistent with what has been observed among males experimentally exposed to different testosterone levels *in utero* (Dean et al. 2012; Kita et al. 2016). Alternatively, it could be that the density of males within the postnatal, preweaning maternal environment led to differences in sperm sex ratios. Under this scenario, exposure to more brothers within more male‐biased litters may have altered the development of the testes at this early life stage, resulting in a lasting effect on spermatogenesis. We speculate that differences in testosterone levels of the individual in response to the postnatal, preweaning environment could account for this outcome. Certainly, elevated testosterone levels is likely to be the underlying mechanism responsible for the development of enhanced body size, testes size, and AGD (Zielinski and Vandenbergh 1993), which, among mammals, are commonly observed responses to increased competition within the postmaternal environment (Ramm et al. 2015; André et al. 2018; Firman et al. 2018; Fisher et al. 2018). Adding to this, our current investigation suggests that differences in testosterone levels of individuals exposed to different postmaternal social regimes may also lead to variation in sperm sex ratios. In our future research, we will elucidate the role that testosterone plays in influencing sperm sex ratios at different developmental stages (i.e., from *in utero* to sexual maturity).

It has been shown that a mother's experience of the environment, which can lead to variation in maternal growth, condition, or physiological state, may be reflected in the phenotype of her offspring (Mousseau and Fox 1998), and it has been suggested that such maternal effects may constrain sex allocation through physiological changes in response to the gestational environment (Edwards et al. 2016b). Our result of a significant effect of the maternal environment on sperm sex ratio establishes an intriguing possibility of transgenerational constraints on offspring sex allocation. There is evidence that maternal stress and testosterone levels can influence primary sex ratios at conception (Navara 2010; Merkling et al. 2018). Female house mice are known to bias offspring sex ratios according to the social conditions that they experience during sexual development, with the underlying mechanism being linked to elevated corticosterone levels (R.C. Firman unpubl. ms.). Consequently, maternal “control” over embryo sex ratios, and potentially intrauterine positioning, may be an effective means by which females prepare their offspring for future environmental conditions (adaptive maternal effect) (Mousseau and Fox 1998). For example, when reared under high male density conditions female house mice produce female biased litters (R.C. Firman unpubl. ms.), potentially resulting in sons that produce lowered sperm sex ratios, an adaptive outcome that aligns with local mate competition theory. Thus, our result may represent a novel finding of a maternal effect on sperm sex ratios (although see Edwards et al. 2016b for a study on lab mice that reported no effect). Importantly, our investigation on wild house mice provides compelling evidence that the maternal environment influences sperm sex ratios, which opens up new avenues for discovery in the study of male‐driven sex allocation.

## Conclusions

Our findings show that male competition during development can influence plastic responses in sperm sex ratios, and highlight the potential that these adjustments function as a mechanism of male‐driven sex allocation. We found that exposure to high male density in the maternal and postmaternal environments leads to an increased production of Y‐CBS. In agreement with previous studies, our data also showed that the male phenotype “prepares” for reproductive competition following exposure to rivals in the postmaternal environment. Our future research will specifically test the male fertility hypothesis of paternal sex allocation and determine whether testosterone is the underlying mechanisms responsible for the observed responses in sperm sex ratios. The sperm sex ratios reported here are all male biased, which may be a feature of the source population, the consequence of natural variation among individuals (Edwards et al. 2016a), or due to the general laboratory conditions under which our experimental males were maintained. In nature, males in good condition have been shown to favor the production of sons (Gomendio et al. 2006; Douhard et al. 2016). Therefore, the average elevated increase in Y‐CBS production that we observed in our wild house mice held in captivity may be a consequence of unrestricted access to nutrition and water. To address this, our future investigations will also focus on variation in sperm sex ratios in natural populations of house mice that differ in social conditions.

Associate Editor: R. Snook

## Supporting information


**Figure S1**. Postmaternal social environment manipulation.
**Figure S2**. The relationship between litter size and litter sex ratio.
**Figure S3**. The postmaternal social environment did not influence epididymal sperm concentration in house mice.

**Table S1**. Full linear mixed models (LMMs) on the effect of the social environment and litter sex ratio on anatomical traits of male house mice.
**Table S2**. Full generalized linear mixed model (GLMMs) using a binomial distribution of errors to assess the effect of the social environment and litter sex ratio on sperm sex ratios of house mice.
