# Supplementary material for: Exposure to male‐dominated environments during development influences sperm sex ratios at sexual maturity
Source: Evol Lett. 2019 Jun 27;3(4):392–402. doi: 10.1002/evl3.123 (PMC6675145; doi:10.1002/evl3.123)
Supplement: Supplementary file 1 — Figure S1. Postmaternal social environment manipulation. Figure S2. The relationship between litter size and litter sex ratio. Figure S3. The postmaternal social environment did not influence epididymal sperm concentration in house mice. [file EVL3-3-392-s001.docx]

Additional Information

**Exposure to male-dominated environments during development influences sperm sex ratios at sexual maturity**

Misha D. Lavoie^1,2^, Jamie N. Tedeschi^1,3^, Francisco Garcia-Gonzalez^1,4,5^ and

Renée C. Firman^1,6^

*^1^Centre for Evolutionary Biology, School of Biological Sciences (M092), The University of Western Australia, 35 Stirling Highway, Crawley WA 6009, Australia*

*^2^Email: 21833982@student.uwa.edu.au*

*^3^Email: jamie.tedeschi@uwa.edu.au*

*^4^Estacion Biológica de Doñana, CSIC, Sevilla, Spain*

*^5^Email: paco.garcia@ebd.csic.es*

*^6^Email: renee.firman@uwa.edu.au*

**Author for correspondence:** Renée C. Firman

email: [renee.firman@uwa.edu.au](mailto:renee.firman@uwa.edu.au)

phone: +61 (0) 439 099 587

**
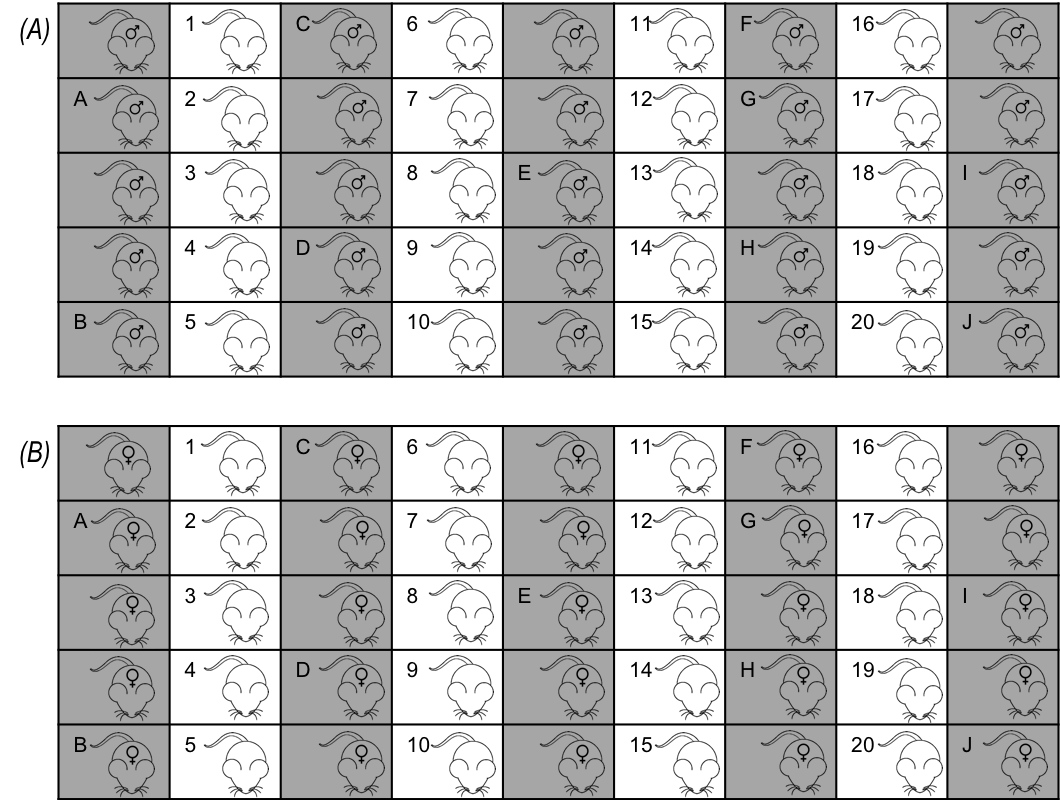
Figure S1.** Post-maternal social environment manipulation. (*A*) The arrangement of the experimental males (white cells) in the high male density treatment in relation to the ten sexually mature (A – J) and 15 equivalently aged non-focal males (grey cells). Twice a week, each focal male was exposed to 15 g of soiled chaff from the ten non-focal sexually mature males (A – J). Once a fortnight each focal male was released into a large, plastic opaque tub containing two of the sexually mature, non-focal males (A – J). The experimental males were periodically exposed to soiled chaff from a sexually mature female to ensure normal reproductive development. (*B*) The arrangement of the experimental males (white cells) in the high female density treatment in relation to the ten sexually mature (A – J) and 15 equivalently aged females (grey cells). Twice a week, each male was exposed to 15 g of soiled chaff from the ten sexually mature females (A – J). Once a fortnight each male was released into a large, plastic opaque tub containing two of the sexually mature females (A – J). During the experiment, the males were rotated across the different rack positions (within treatments).

**
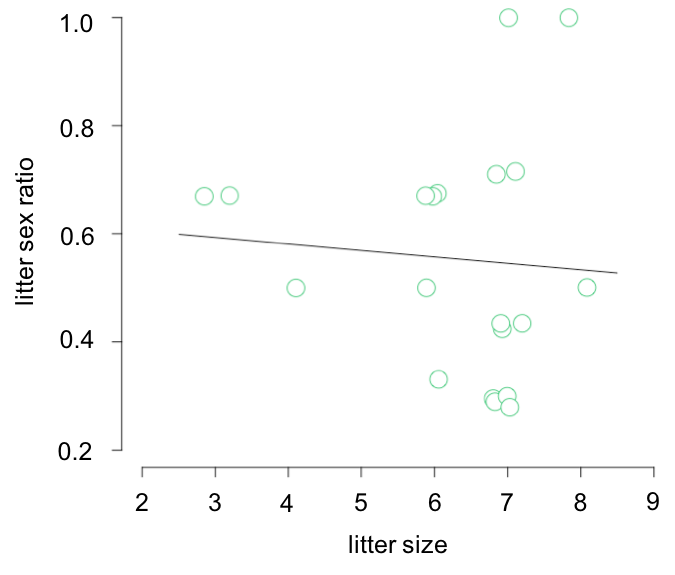
**

**Figure S2.** The relationship between litter size (*i.e.,* the proportion of male offspring) and litter sex ratio. Regression analysis: *F*_1,18_ = 0.109, *P* = 0.744, *r* = 0.077.

**
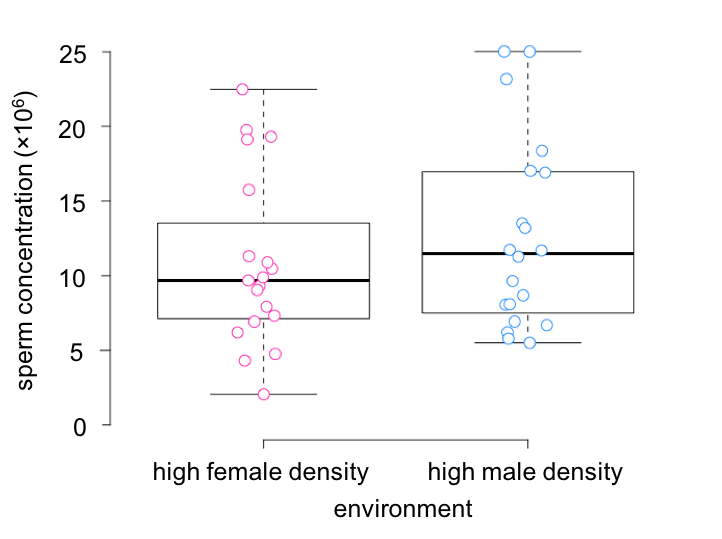
**

**Figure S3.** The post-maternal social environment did not influence epididymal sperm concentration in house mice.
